# Pharmacist dispensing of the abortion pill in Canada: Diffusion of Innovation meets integrated knowledge translation

**DOI:** 10.1186/s13012-021-01144-w

**Published:** 2021-08-03

**Authors:** Sarah Munro, Kate Wahl, Judith A. Soon, Edith Guilbert, Elizabeth S. Wilcox, Genevieve Leduc-Robert, Nadra Ansari, Courtney Devane, Wendy V. Norman

**Affiliations:** 1 Centre for Health Evaluation and Outcome Sciences, Providence Health Care Research Institute, Vancouver, British Columbia Canada; 2 Department of Obstetrics & Gynaecology, Faculty of Medicine, University of British Columbia, Vancouver, British Columbia Canada; 3 Faculty of Pharmaceutical Sciences, University of British Columbia, Vancouver, British Columbia Canada; 4 Department of Obstetrics, Gynaecology and Reproduction, Laval University, Quebec City, Quebec Canada; 5 School of Population and Public Health, University of British Columbia, Vancouver, British Columbia Canada; 6 Faculty of Medicine, University of British Columbia, Vancouver, British Columbia Canada; 7 Faculty of Health Sciences, Simon Fraser University, Burnaby, British Columbia Canada; 8 School of Nursing, University of British Columbia, Vancouver, British Columbia Canada; 9 Department of Family Practice, University of British Columbia, Vancouver, British Columbia Canada; 10 Faculty of Public Health and Policy, London School of Hygiene and Tropical Medicine, London, UK

**Keywords:** Mifepristone, Pharmacists, Canada, Abortion, induced, Primary Health Care, Diffusion of Innovation

## Abstract

**Background:**

Since Canadian drug regulatory approval of mifepristone for medical abortion in 2015 and its market availability in January 2017, the role of pharmacists in abortion provision has changed rapidly. We sought to identify the factors that influenced the initiation and provision of medical abortion from the perspectives of Canadian pharmacists, bridging two frameworks — Diffusion of Innovation in Health Service Organizations and integrated knowledge translation.

**Methods:**

We conducted one-on-one semi-structured interviews with pharmacists residing in Canada who intended to stock and dispense mifepristone within the first year of availability. Our data collection, analysis, and interpretation were guided by reflexive thematic analysis and supported by an integrated knowledge translation partnership with pharmacy stakeholders.

**Results:**

We completed interviews with 24 participants from across Canada: 33% had stocked and 21% had dispensed mifepristone. We found that pharmacists were willing and able to integrate medical abortion care into their practice and that those who had initiated practice were satisfied with their dispensing experience. Our analysis indicated that several key Diffusion of Innovation constructs impacted the uptake of mifepristone, including: innovation (relative advantage, complexity and compatibility, technical support), system readiness (innovation-system fit, dedicated time, resources), diffusion and dissemination (expert opinion, boundary spanners, champions, social networks, peer opinions), implementation (external collaboration), and linkage. Participants’ experiences suggest that integrated knowledge translation facilitated evidence-based changes to mifepristone dispensing restrictions, and communication of those changes to front line pharmacists.

**Conclusions:**

We illustrate how Diffusion of Innovation and integrated knowledge translation may work together as complimentary frameworks for implementation science research. Unlike in the USA, UK, and other highly regulated settings globally, pharmacists in Canada are permitted to dispense mifepristone for medical abortion. We contribute to literature that shows that mifepristone dispensed outside of hospitals, clinics, and medical offices is safe and acceptable to both patients and prescribers. This finding is of particular importance to the current COVID-19 pandemic response and calls for continued and equitable access to abortion care in primary practice.

**Supplementary Information:**

The online version contains supplementary material available at 10.1186/s13012-021-01144-w.

Contributions to the literature

- We demonstrate how to bridge Diffusion of Innovation and integrated knowledge translation constructs to investigate implementation of a pharmacological intervention, using the case of the abortion pill, mifepristone.
- Our analysis of interviews with pharmacists indicates that diffusion of information about the medication through organizations and champions was key to implementation.
- Integrated knowledge translation practices can leverage communication with organizations and champions, further facilitating implementation.

## Background

Canada is one of the first pharmaceutically regulated countries in the world to approve pharmacists’ dispensation of mifepristone, the medical abortion pill, directly to patients [1, 2]. This task sharing between pharmacists, as experts in medication stocking, dispensing, and counselling, and prescribing healthcare providers is considered preferable for the provision of prescription medications [3–5]. In provinces like Quebec and according to its physician’s code of ethics, physicians cannot stock or dispense most prescription medications, including mifepristone, to avoid conflict of interest; only pharmacists can [6–8]. This evidence- and ethically-based approach stands in contrast to more restrictive first trimester medical abortion regulations in the USA and UK, where mifepristone is dispensed to patients by the authorized prescriber [9, 10]. Safe and effective task sharing to dispense medications is within the pharmacist scope of practice and offers an opportunity to improve access to care [11].

Historically, medical abortion in Canada could be provided only through off-label use of methotrexate and misoprostol prescribed by physicians in private, community, or hospital-based specific abortion clinics, and more than 95% of abortion care was surgical [12]. In 2015, mifepristone was approved in Canada and first became commercially available in January 2017 but was subject to restrictive requirements (Table 1). These included dispensation to patients directly by a physician with observation of the initial mifepristone dose, mandatory training and certification of prescribing physicians and pharmacists, and registration of prescribing physicians and pharmacists with the manufacturer [13, 14]. By November 2017, each restriction was removed, paving the way for pharmacists to dispense mifepristone and providing a test-case for task sharing of medical abortion services in routine primary care. Pharmacist attitudes toward participation in emergency contraception [15] and other family planning care have been found to be positive [4], indicating potential openness to participating in abortion care.

**Table 1:** Changes to Health Canada restrictive measures for mifepristone-misoprostol medical abortion

Topic	Change	Date changed
**Observed ingestion**	Removed requirement for observation of mifepristone ingestion. The patient can take the medication where and when they choose.	Oct 2016
**Training**	Removed requirement for training for pharmacists.	May 2017
**Training**	Removed requirement for training for prescribers.	November 2017
**Consent form**	Removed requirement for a manufacturer consent form to be signed by the patient.	November 2017
**Registration**	Removed requirement for registration of prescribers or pharmacists with the manufacturer.	November 2017
**Dispensing**	Mifepristone can be dispensed directly to patients by a pharmacist or prescribing health professional, rather than the original requirement that a physician must dispense directly to the patient.	November 2017
**Gestational age**	Mifepristone-misoprostol may be used up to 9 weeks (63 days) from last menstrual period, rather than the original 7 weeks (49 days).	November 2017
**Ultrasound**	Removed requirement for mandatory ultrasound prior to prescribing.	April 2019

Source: Munro et al. [13]

**Fig. 1:**
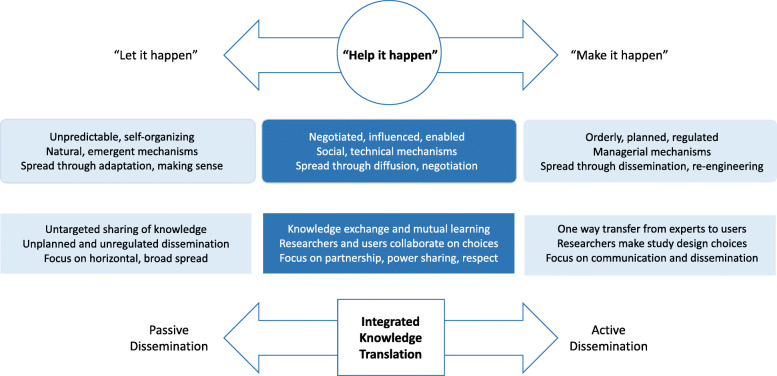
A conceptual basis for knowledge spread where Diffusion of Innovation meets integrated knowledge translation. Adapted from Greenhalgh et al. [19] and Bowen and Graham [21]

While pharmacists have the potential to facilitate rapid community-based access to the medication and to streamline reimbursement mechanisms for patients [5], implementing new pharmaceutical therapies like medical abortion can be a complex process. Diffusion of Innovation theory can be a helpful framework for investigating the constellation of factors that influence real-world implementation in pharmaceutical practice [16–18]. The theory posits that implementing an innovation (e.g., mifepristone) depends on its simplicity and trialability, its benefits and advantages relative to what was previously used, and its fit with adopters’ values, needs, and tasks [19]. Implementation also depends on the abilities and willingness of the adopter (e.g., pharmacists), the size and readiness of their organizations, and the support and resources offered by others in and outside the health care system. Implementation efforts may exist on a continuum from highly managed (“make it happen”) to flexible and adaptive (“let it happen”) [19]. For systems-level challenges involving stigmatized health services, like the implementation of mifepristone abortion care in Canada, Greenhalgh and Papoutsi argue that “ecological and social practice perspectives” like Diffusion of Innovation are particularly appropriate as they follow the logic of complex systems which are characterized by unpredictability, interdependencies, and self-organization [20]. One additional strategy that can “help it happen” and facilitate self-organization through relationships and sensemaking is integrated knowledge translation (KT), the process of partnering with knowledge users at all stages of an implementation study [21]. The collaboration can include co-developing the research question, making shared choices about study design, partnering to design study tools and participate in data collection, and interpreting and disseminating results together. In integrated KT, there is an implicit understanding that knowledge users and researchers bring complmentary contextual and methodological expertise to the process [22]. The continuous collaboration involved in integrated KT — characterized by social interaction and negotiation to enable spread of knowledge — closely reflects Greenhalgh’s “help it happen” approach to diffusion of innovations in health care (see Figure 1).

The present research was part of a larger mixed-methods investigation [23]. In the main study, we asked the following questions: *What are the factors that influence successful initiation and ongoing provision of medical abortion services among health professionals, and how do these relate to health policies, systems, and services, and to abortion service access throughout Canada?* For the present analysis, we focused on the first question involving the identification of factors that influence the initiation and provision of medical abortion from the perspectives of Canadian pharmacists. We demonstrate how we operationalized two complimentary approaches that embrace a complexity lens — Diffusion of Innovation theory and integrated KT.

## Methods

This study was part of a national mixed-methods programme of research designed to characterize and facilitate the implementation of mifepristone medical abortion between 2015 and 2019 [23]. The research was informed by an integrated KT approach premised on the understanding that research is more relevant and useful when knowledge users are equal partners in the work [24, 25]. Consequently, pharmacist stakeholders were members of the research team and contributed to study design, recruitment, interpretation, and dissemination. As planned with our integrated KT approach, we engaged in monthly feedback meetings to exchange results in progress to stakeholders, guided by principles of sensemaking [26, 27] — the process through which people assign meaning to experience. Our sensemaking sought to understand how mifepristone implementation unfolded and exchange real-time insights to encourage evidence-based practice and policy action that would facilitate implementation.

The theoretical framework that guided our study was Diffusion of Innovation in Health Service Organizations described by Greenhalgh and colleagues, which includes six broad constructs representing 58 dimensions [19]. Although Diffusion of Innovation captures the interdependence of individual, organizational, and contextual factors affecting implementation, its complexities are difficult to capture in applied research [28, 29]. We therefore adapted Cook and colleagues’ operationalization of the constructs, which has been applied in previous qualitative investigations [28, 30–34]. Our use of integrated KT further acted to support each researcher and knowledge user on the team to gain a shared understanding of this theory and co-create a study design guided by the framework.

### Participants and recruitment

Participants eligible for this study were pharmacists residing in Canada who intended to stock and dispense mifepristone within the first year of availability and could speak English or French. Participants were recruited by email from a list of pharmacists who had consented to be contacted for an interview in a previous survey circulated through the Canadian Abortion Providers Support community of practice [35]. We purposefully sampled for a diversity of characteristics relevant to participation in abortion care (e.g., previous experience in family planning, geographic region, gender, age, timing of adoption of this new practice). We continued sampling until we had satisfied key markers of saturation: the characteristics were well-represented; additional interviews were consistent with previous data; no new themes were identified in analysis; and each theme was demonstrable within the sample [36, 37]. Characteristics of participants were documented for sampling and analysis but are not reported in the study in order to maintain participant anonymity.

### Data collection

We conducted one-on-one telephone interviews with participants between June 2017 and February 2018, which allowed us to characterize uptake of mifepristone among pharmacists before and after the removal of restrictions on this medication in November 2017. The interviews proceeded according to a semi-structured interview guide (see Additional file 1) informed by Cook and colleagues’ operationalization of the Diffusion of Innovation constructs and developed and pilot tested with an expert panel of clinicians and researchers [28]. Senior health services researchers (SM, EG) and trainees oriented in the study procedures (CD, GL-R) conducted the interviews, which were audio-recorded. The study lead (SM) was a qualitative researcher while all other team members had clinical backgrounds (family medicine, obstetrics and gynaecology, public health, pharmacy, nursing). Interviewers engaged in reflexive practice by considering the relative status, power, and comfort of participants throughout the data collection process, as well as how their training and background may influence their interpretation of the data. Participants provided verbal consent at the beginning of the interview.

### Data analysis

The interviews were transcribed, de-identified, and assigned a numeric identifier (e.g., Participant 001). We translated the French transcripts to English. Led by a member of the study team with expertise in qualitative research (SM), three trainees (NA, EW, KW) conducted a reflexive thematic analysis of the data according to the flexible approach described by Braun and Clarke [38–40]. This approach was selected as it focuses on researcher subjectivity and knowledge as constructed, situated, and contextual. We were not seeking a single truth but rather multiple perspectives that capture the complexity of implementation of mifepristone, consistent with our theoretical framework, Diffusion of Innovation. We familiarized ourselves with the data by reading the transcripts as a whole and noting initial impressions. We adopted a complexity standpoint and approached our analysis with the belief that implementation is more than the sum of its parts; it is characterized by the dynamic interplay between elements and relationships. Thus, our team engaged in multiple stages of analysis; we went beyond coding and categorization to also engage in mapping processes and relationships. Analysis of interview transcripts involved four iterative steps:

1. Inductively identifying contextual codes related to our research question;
2. Refining codes through iterative analyses that considered patterns across participant data; relationship among individual, organizational, and system-level themes; conflicting themes; and the observed relevance of themes to the research question;
3. Identifying individual, organizational, and system processes (including patterns, relationships, and interactions) between the codes; and
4. Deductively mapping the results of this analysis (codes, patterns, and relationships) to the Diffusion of Innovation framework through iterative team-based workshopping sessions and during manuscript preparation.

Discrepancies that arose were resolved by consensus among the study team. Strategies to support the rigour of our analysis included constant comparison, audit trails, and meetings with pharmacist stakeholders to discuss and contextualize results in progress.

## Results

### Participants

We conducted 24 one-on-one interviews with pharmacists involved in the dispensing of mifepristone medical abortion services in Canada. All participants were volunteers who consented to participate and completed their interview. On average, each interview lasted 45 min. Of the participants, 33% had stocked and 21% dispensed mifepristone; of the remaining participants, all but one intended to distribute mifepristone in the future. Participants were geographically distributed, with 46% from western provinces (British Columbia, Alberta, Saskatchewan, Manitoba), 38% from central provinces (Ontario, Quebec), and 17% from an Atlantic province or Territory (New Brunswick, Yukon).

**Fig. 2:**
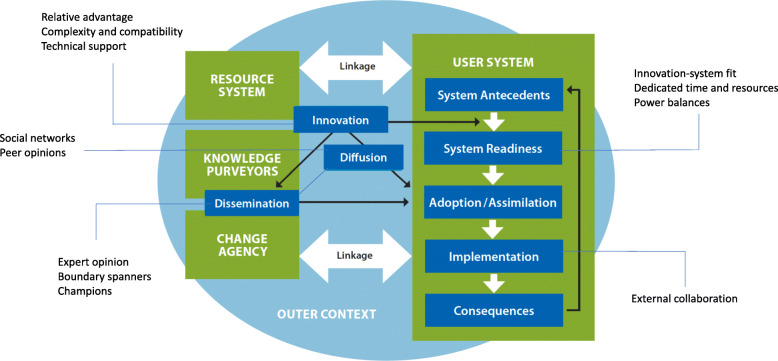
Determinants of diffusion of innovations in health service delivery organizations, adapted from Greenhalgh et al. [19]. Source: Norman et al. [23]

Five broad Diffusion of Innovation constructs, comprising a total of 13 Diffusion of Innovation dimensions, emerged as important to pharmacist participation in medical abortion care (see Figure 2). These included the *innovation* (relative advantage, complexity and compatibility, technical support), *system readiness* (innovation-system fit, dedicated time and resources, power imbalances), *diffusion and dissemination* (expert opinion, boundary spanners, champions, social networks, peer opinions), *implementation* (external collaboration), and *linkage*.

### The innovation: mifepristone

#### Relative advantage

Relative advantage refers to the perception that the innovation is superior to existing alternatives. Participants agreed that mifepristone carried clear advantages related to increased reproductive choice for people seeking abortion care, more equitable access for people living in rural areas without local surgical abortion care, privacy and convenience for those seeking to have an abortion at home, a less invasive experience than surgical care, and greater effectiveness compared with previous off-label medical abortion regimes. As one participant from a western province said:

The huge thing is that most of the patients, especially if they are coming from rural areas, if any pharmacies were there, they don’t have to travel five, six hours to a city like this to actually have it even done because it’s not surgical. It’s just a medication that can be dispensed. In addition, of course, the success rate is great. It’s fairly close to sort of the surgical component, but it doesn’t carry some of the associated risks … Thirdly, it provides patients not only accessibility, but a bit of privacy as well, which is actually another thing that I think we’ll need to discuss. Privacy and accessibility and then the rate of success, I think, are probably the three biggest advantages to it. (003)

The related Diffusion of Innovation dimensions of risk and assessment of implications were relevant to how participants viewed potential disadvantages of mifepristone. A few participants raised the possibility of patients having complications in the community, without a guaranteed way to follow up. Others pointed out that reimbursement for the cost of mifepristone was a challenge both for patients paying out of pocket (before reimbursement by public and private insurance could be settled) and for pharmacists stocking the medication without a sense of consumer demand. The concern about costs was articulated by an urban participant from a central province who explained:

It does cost $300. From an inventory standpoint, we can’t have shelves and shelves of it. We keep a minimum stock. We haven’t been able to assess trends. That’s what we use to stock the pharmacy. We look at trends over weeks, over months to see how much of this do we dispense. It’s still relatively new, and you’ve only dispensed it to one patient. You can only keep two boxes. From that point, it’s just lack of data might impede on ability to stock it. (020)

#### Complexity and compatibility

Many participants perceived that dispensing mifepristone was no different from other medications — it had similar complexity and compatibility. The complexity of an innovation depends on how difficult it is to use while its compatibility relates to the degree of alignment with system and user values, needs, and experiences. For example, one participant from a western province explained:

The minute we receive the prescription, it’s as similar to any other prescription. Some of the medication, we have to order for the next day, and then we arrange for the patient to come and sit with one of our pharmacists to talk about it. Similar to any new medication for any other medical condition. (017)

Specific concerns about the complexity of mifepristone mostly related to counselling, which all participants agreed should be comprehensive. The notion that counselling complicated care appeared to depend on the degree to which the pharmacist felt equipped to discuss the potentially sensitive topic of abortion. For instance, one participant from an Atlantic province explained,

Obviously, the person who is seeking … a very sensitive product, so it does require maybe a greater level of empathy or that sort of emotional part that goes along with it as well. Definitely, I feel it’s in the pharmacy’s scope. I feel it’s in my scope, but I feel like I need a higher level of kind of effort that goes into it because there may or may not be an emotional part as well. (001)

Another participant from a central province (021) pointed out that although dispensing mifepristone might be time-consuming, there would be a low volume of prescriptions in their rural town and mifepristone would have a limited impact on their workload and workflow. Notably, pharmacists who had dispensed mifepristone articulated less concern about counselling, as one participant described,

After the first maybe two or three patients I dealt with, it became fairly sort of standard, easy, and I felt a lot more comfortable in terms of dispensing it. (003)

#### Technical support

Participants articulated that their mifepristone practice was supported by adequate training, namely the national online training course (Society of Obstetricians and Gynaecologists’ Training on Medical Abortion) that was originally required for any pharmacist involved with mifepristone provision. Overall, participants felt that the course had an appropriate level of difficulty and was consistent with other training they had completed. Some participants noted that the length of the training was a barrier and that a course specifically designed for pharmacists would be more professionally relevant. As one participant from Quebec said, “Three and a half hours is far too long for the impact it will have on our practice. On the other hand, if there was a specific module on pharmacology, maybe we could use it” (Q05). Others mentioned that the limitation of the free, unaccredited version of the training programme, versus the paid version providing continuing education credits, was a potential barrier.

Participants described drawing on various resources for ongoing support, including sponsored in-person training from their pharmacy chain and step-by-step guidance from professional organizations. However, several participants felt that they would benefit from additional support, including updates on coverage available for patients, lists of local prescribers, and algorithms or summary sheets to “make sure certain points are discussed and we aren’t missing anything and everything is documented properly” (010).

Anticipating that prescribers and pharmacists would need additional technical support for initiating this new practice, our research team was part of a national effort to create a community of practice, the Canadian Abortion Providers Support (CAPS) platform [35]. A few participants noted in particular that registering with the CAPS community, weekly online eye-catching bulletins, and receiving monthly emailed updates from this website provided them with ongoing support and information.

### System readiness for mifepristone

#### Innovation-system fit

For some participants, there was a poor fit between community pharmacy and mifepristone dispensing when the initial restrictions were in place. As one participant noted, the mandated training and the patient consent form added time and expense to workflow: “It was very challenging to start … because you almost had to go through 50 hoops … It used to be that we had to give it to [physicians] to give to [patients]. It was like it was acid or something ... Mandated health professional training is no longer required. At the time, I had to get past a test to be able to order it from [the manufacturer]” (018). The initial regulation that physicians should dispense the medication was perceived to be at odds with scope of practice for the two professions. As one participant pointed out,

Physicians, I believe that they are more focused on diagnosis and deciding what medication to use, but when it comes to the medication itself, I think it’s best to get it from the pharmacy, from a pharmacist, because pharmacists, I believe that they are more knowledgeable when it comes to medications (008).

Overall, participating pharmacists were either unaware of the restrictions or did not find them to be an issue. One person who was interviewed after all restrictions were removed in November 2017 reflected on the early days of mifepristone availability: “I know at that time not every pharmacy was able to dispense it. You had to take a course and register with the company and whatnot. That was before they removed that barrier” (020).

#### Dedicated time and resources

Because counselling was perceived to be an important component of the dispensing process, system readiness for mifepristone was enhanced when the pharmacy had a private counselling room that would allow for consultations with patients. Participants had varying perspectives about the cost of providing counselling, with a few highlighting a need for this service to be reimbursed in addition to the dispensing fee. The time when mifepristone counselling was required was also a factor, with some participants raising the concerns about whether patients could be accommodated during peak hours. However, others pointed out that the need for counselling was not unique to mifepristone and that the need to triage patients during high-volume times was “just retail pharmacy” (020). The tension between workload and provision of care was articulated by one participant from Quebec who explained the following:

In the pharmacy, sometimes we have many things to do all at once, so we're really overloaded at times and sometimes stressed. But we’re still going to take the necessary time with someone for this type of intervention … So, while it’s stressful when there's something new, at the same time, it's not negative either. (Q04)

#### Power balances (supporters vs. opponents)

A potential barrier to mifepristone uptake was difference in support toward abortion care within pharmacy settings, including conscientious objectors who opposed implementation, and the relative power of the individuals involved in making implementation decisions. The majority of participants expressed pro-choice attitudes but observed ethical objections to abortion care around them, including among colleagues and in their community. Only two participants expressed personal qualms about the ethics of abortion, but they focused on their professional responsibility to provide care saying, for example,

I was really thinking about it. I was contemplating about it for a long time before continuing the course, but I was actually thinking the best probably that I can give to the patient who has a prescription for it would be full information of the product. (008)

Other participants described how even if their pharmacy stocks and dispenses mifepristone, individual pharmacists would have the ability to decline a patient’s prescription. One participant illustrated how individual pharmacists would have the power to oppose abortion care:

There’s a couple of colleagues that are fairly religious … They wouldn’t be comfortable being involved in that process as far as I’m aware. Then that would be a little bit of a barrier, so if they were the only one on the shift and the patient came to them, they would have to send them to another pharmacy. (010)

Other participants also raised this possibility and pointed to mitigating factors. They perceived, for instance, that colleagues with anti-choice views were rare. If a patient presented to an unsupportive pharmacist, it was likely that another, supportive team member would be available for the patient. This was exemplified by one participant who described how one of four team members refused to provide mifepristone,

She represents fifteen hours a week ... It's not a big challenge to work with the limitations brought by her conscientious objection to the dispensation of the service, and besides, one works around her skills and comforts to adjust. (Q03)

Notably, although almost all participants described their professional communities as pro-choice, social norms about abortion may have prevented some participants from establishing external collaboration. For example, one participant from an Atlantic province commented, “It’s not something that’s talked about a lot” (014).

The only reported instance in which anti-choice attitudes within a team significantly hindered adoption of mifepristone was when management at an independent pharmacy asked the team to come to a consensus about whether or not to provide abortion medications:

I think our team views it as a risky subject because it is not only the people who are receiving but also the team providing it, if they have an ethical dilemma or they have a belief that we shouldn’t be providing this … I was told that we all have to decide as a team if this is ethical and comfortable for us. (002)

### Diffusion and dissemination of mifepristone: “Help it Happen”

Effective communication about mifepristone — what it is, how to dispense it, and what federal restrictions were in place — helped to spread information about medical abortion among pharmacy practices in Canada. Participants’ experiences suggested that spread was primarily through active dissemination, where communication was planned through formal professional channels by trusted, influential experts, and authorities.

#### Network structures

The diffusion and dissemination of mifepristone practice was facilitated by two types of networks. Vertical networks with professional organizations and colleges disseminated information about the easing of restrictive measures and authoritative decisions, like announcements of public coverage for the pill. Horizontal social networks with peers and champions helped to spread information and supported mifepristone distribution as a routine pharmacy practice. Both network structures worked to normalize mifepristone as part of pharmacist scope of practice.

Most participants described receiving links to educational material from professional organizations such as the Canadian Pharmacists Association and provincial College of Pharmacists as well as from their corporate chain (e.g., e-bulletins or an on-site consultant). In addition to raising awareness of the training programme and other educational resources, these interactions helped normalize the practice of dispensing mifepristone even before the change in government regulations. As one participant described,

My sense from [the College of Pharmacists] is that they want you to do whatever is right for the patient whether the monograph says it should go through the pharmacy or not. I think they feel that the pharmacists play a role in dispensing this product and not just dispensing the product but taking care of the patient. I think they would wholeheartedly support this going through where the patient gets prescription. (001)

The effect of this communication on normalizing abortion care as part of the pharmacy scope of practice was very important for some participants. For example, one pharmacist who was resistant to supporting abortion for religious reasons remarked,

The moment I received an e-mail from [my professional organization], it made me feel that eventually all pharmacists would be dispensing it, and all pharmacists are obliged to at least be knowledgeable about the product to help moms in case they would have the prescriptions. (008)

Similarly, corporate offices were described as taking steps to keep pharmacists up-to-date on regulatory changes and to make mifepristone a routine component of policies and procedures. While this vertical network communication was important for raising awareness and acceptance of mifepristone dispensing, several participants commented that it was too infrequent or inaccessible. As one participant pointed out, “A lot of the times, you’ll learn stuff and start getting stuff, but if you don’t kind of implement it or take initiative right away, things don’t just happen” (009). This was echoed by another participant, who said, “A one-time letter is not going to make it happen. Like, I got this one letter from the college … it’s a lengthy letter. It’s huge. It’s not appealing for people who only have a minute to read the e-mail” (002). Receiving brief, regular updates thus appeared vital to support implementation and routinization of the pharmacy practice.

Champions external to the team who held regional leadership roles also played a diffusion and dissemination role for some participants. For example, one participant explained that “Dr. [name redacted] who is next door, yeah, she’s played a big role moving this forward and getting information out there for training with other professionals, pharmacy or physicians, out there looking for more information” (012).

In most cases, managers, pharmacists, and assistants within teams initiated informal communication through their horizontal peer networks about training opportunities and had discussions about who on the team would and would not be comfortable participating in abortion care. Several participants also had or planned to reach out to prescribers in the community to inform them that mifepristone was available at their pharmacy. Examples of passive communication were less common among participants, but one participant was engaged in a Facebook group for pharmacists that shared information about mifepristone.

### Implementation of mifepristone in pharmacy practice

#### External collaboration

Participants’ experiences suggest that communication with prescribers was the most important factor for pharmacists to decide whether to stock mifepristone in their pharmacy dispensary. External collaboration depended on whether local prescribers were aware of mifepristone for medical abortion, willing to prescribe the medication, and familiar with the community pharmacies in their area that were stocking the medication. Some pharmacists had strong ongoing collaborative physician-pharmacist relationships that supported seamless implementation. As one participant described,

A group of us – two pharmacists, the nurse practitioner who works in the sexual office clinic, and a couple of family doctors – we got together, talked about how we were going to do it locally … I think we’ve already helped quite a few women, and the process has been – with a few little tweaks along the way – it’s been very, very smooth. (004)

Pharmacists perceived that mifepristone medical abortion might be complex for prescribers, infrequent in their population, or incompatible with their values. For example, one participant said,

I haven’t talked to any of the local physicians personally, but I don’t expect any of them would be uncomfortable. That being said, I also think a lot of them would refer. I think they’re comfortable with the drug itself but perhaps uncomfortable prescribing, were I to wager a blind estimate just based on their prescribing histories (015).

In some cases, sustainable implementation of mifepristone depended on the prescriber being willing to send patients to the participant’s pharmacy for mifepristone prescriptions. One participant described reaching out to a high-volume abortion provider to let them know about the availability of mifepristone in that pharmacy. Initially, the participant described, “She and I had a great working relationship because we figured out essentially what information we gave to the patient, agreed upon the process, what form she was going to give to the patients to bring to me, and also if there were any issues at all, for me to communicate with her” (003). However, when a pharmacy closer to this prescriber’s clinic began to dispense mifepristone, the participant stopped receiving clients, reflecting, “I’ve sort of run out of options as to how do I go about dispensing it or getting physicians to actually send people this way” (003).

These collaborative partnerships were rare and the dominant sentiment from participants was “physicians don’t know that we can provide it … that’s why we haven’t seen it yet” (009). These pharmacists described having no or few conversations with prescribers about mifepristone. They perceived that their experiences of low consumer demand (i.e., few mifepristone prescriptions received at the pharmacy) was due to prescriber barriers such as lack of familiarity with mifepristone, lack of awareness that primary care physicians and nurse practitioners could provide medical abortion, and perhaps an unwillingness to provide this care.

### Interaction between domains and over time

The notion of time recently has been integrated into Diffusion of Innovation models to account for the dynamic changes that occur over an implementation journey, and the concomitant need to adapt an innovation in response to feedback [41]. The experiences of study participants indicate that relationship building and feedback over time, including integrated KT activities like our CAPS community of practice, were a key facilitator for mifepristone implementation. Soon after mifepristone was made available in 2017, Health Canada quickly updated the product label to enable usual and customary pharmacist dispensing for this medication. This change was one of the first made by federal decision makers in their removal of restrictive measures. Participants perceived it was communicated efficiently through pharmacy licencing colleges and professional organizations. Strong connections between pharmacists and their professional and corporate organizations (vertical network structures) supported prompt communication about changing training requirements and Health Canada measures for dispensing of mifepristone. Over time, as the innovation-system fit became more compatible, the challenge shifted from *system readiness* to *adoption* in practice, and lack of external collaboration became the pressing issue. Weak interprofessional connections with local prescribers meant pharmacists who intended to practice had limited to no prescriptions arriving at their pharmacy. Developing these collegial professional relationships where none had previously existed was a time-consuming endeavour that required pharmacists to become change agents. As one participant described, “I called to let [the physician] know that, ‘You know what? This is a new drug that just came out in the market. I am one of the pharmacies’” (003).

## Discussion

Our results suggest that pharmacists from across Canada were willing and able to integrate medical abortion care into their practice and those who had initiated this new clinical practice area were satisfied with their ordering of the medication and the dispensing and clinical counselling experiences. These results illustrate how the first year of implementation of mifepristone medical abortion was characterized by the uncertainty of changing restrictive measures and continuous reinvention through self-organization to bring mifepristone dispensing in line with usual practice. Our approach demonstrates how to operationalize the Diffusion of Innovation framework in the context of an integrated KT study and provides a case example of how use of these complimentary approaches may accelerate policy changes and facilitate implementation of a pharmaceutical innovation.

Our thematic analysis indicated that several key Diffusion of Innovation constructs impacted uptake of mifepristone dispensing. Pharmacists perceived that mifepristone would benefit their patients and, especially after the removal of numerous initial Health Canada restrictions, felt that routine patient counselling was unlikely to disrupt clinical practice. At an individual level, pharmacists agreed that providing the gold standard medical abortion treatment carried advantages relative to off-label and surgical options. For most participants, providing abortion care was also aligned with personal pro-choice values or a professional commitment to providing well-informed care, although they sometimes perceived unsupportive, anti-choice attitudes among other professionals. Provision of mifepristone was facilitated in workplaces where professional organizations, corporate bodies, and influential individuals actively encouraged implementation. Strong support from professional organizations and continuing education programmes positively impacted adoption of mifepristone in the community pharmacy setting. Nevertheless, incorporation of mifepristone ordering, stocking, dispensing, and counselling were contingent on the community pharmacists and store managers in each individual pharmacy location developing collaborative relationships with physicians and nurse practitioners able to prescribe the medication and refer their patients to these specific community pharmacy locations. This collegial relationship between prescribers and community pharmacists has the potential to ensure that the community pharmacy maintains mifepristone supplies, and provides patients with the clinical counselling and support that they require.

Our results also suggest that relationship building and feedback — a “help it happen” approach to Diffusion of Innovation — were key facilitators for mifepristone implementation. Throughout the first year of mifepristone availability, our research team engaged in sensemaking with stakeholders from Health Canada, sharing real-time data from the present study. In turn, Health Canada updated the product label to enable pharmacists to dispense the medication, making it consistent with their usual practice [23, 25]. Pharmacy licencing colleges and professional organizations then communicated these changes to their members, our participants. Our approach demonstrates how integrated KT and Diffusion of Innovation may work together as complimentary frameworks to facilitate uptake of evidence-based interventions in routine practice.

Our findings will also be relevant to researchers involved in large-scale implementation research involving abortion or similarly stigmatized health services. Since there are no legal restrictions on abortion in Canada and restrictions on mifepristone were removed by the Canadian regulatory body in the course of our study, policy barriers had minimal impact on Canadian pharmacists. In the USA, where federal policies are a persistent barrier to pharmacist dispensing, retail pharmacists support the removal of restrictions on dispensing mifepristone [3, 42, 43]. These attitudes are consistent with Australian research in which pharmacists dispensing mifepristone felt it was within their routine practice [44]. Previous research has shown that mifepristone dispensed outside of hospitals, clinics, and medical offices is safe and acceptable to both patients and prescribers [13, 45, 46]. Our dual framework approach, bridging integrated KT with the Diffusion of Innovation framework may be a helpful model for other health care systems. In Australia, our approach is being used and tested through the AusCAPPS Network (The Australian Contraception and Abortion Primary Care Practitioner Support), a community of practice that supports the primary care workforce to deliver evidence-based abortion and contraception care, and feedback real-world practice experiences to policy makers to facilitate practice support [47].

We offer a theory-driven, process-oriented, participatory case study of the Canadian pharmacist experience. One strength of our approach is that data collection took place during the period of 2017 when restrictions on mifepristone were removed. Our study also is strengthened by the inclusion of pharmacy knowledge users in integrated KT, who helped ensure the relevance of the work to pharmacy policy and practice. Our use of Diffusion of Innovation to frame the research facilitated a theory-driven approach and allowed us to explore links between implementation constructs that have been investigated in previous studies. The applicability of the results may be limited by the inclusion of only participants who intended to stock mifepristone. This sample was likely more aware of and open to their potential role in providing the medication and were predominantly pro-choice. Similarly, our findings should be cautiously applied to other national contexts where Diffusion of Innovation constructs may interact differently to affect the implementation of mifepristone in primary care.

## Conclusion

The evidence resulting from the Canadian experience can inform the expansion of safe abortion services through task sharing in other highly regulated settings. We illustrate how pharmacists, as highly qualified and accessible health care professionals, can be willing and capable partners in this care, especially when strong interdisciplinary collaborations are in place. Our study demonstrates how to use integrated KT to operationalize Diffusion of Innovation theory for complex, stigmatized implementation challenges, like abortion care.

## Supplementary Information

**Additional file 1.** Pharmacist Interview Script.

## Data Availability

The datasets generated and analysed during the current study are not publicly available due to individual privacy rights of our participants and as outlined to them during the consenting process.
