# Supplementary material for: Pharmacist dispensing of the abortion pill in Canada: Diffusion of Innovation meets integrated knowledge translation
Source: Implement Sci. 2021 Aug 3;16:76. doi: 10.1186/s13012-021-01144-w (PMC8330203; doi:10.1186/s13012-021-01144-w)
Supplement: Supplementary file 1 — Additional file 1. Pharmacist Interview Script. [file 13012_2021_1144_MOESM1_ESM.pdf]

Pharmacist Interview Script

Mifepristone Implementation Research in Canada (The CART-Mife Study)

1. Demographic questions

"First let's review the demographic information you provided in the survey in case anything has changed. I have a couple of Yes/No questions to begin."

Look first at their responses to the baseline survey. Read demographic section of survey aloud again and record any changes.

#	Question
1a.	In your survey you responded that you DO plan to stock mifepristone at your facility.<br>Is that still your plan?<br>• Yes<br>• No<br>(If yes) Have you begun to stock mifepristone?<br>• Yes<br>• No
1b.	In your survey you responded that you DO NOT plan to dispense mifepristone at your facility.<br>Is that still your plan?<br>• Yes<br>• No<br>(If no) Have you begun to dispense mifepristone?<br>• Yes<br>• No

2. Interview questions

Thank you. I would first like to know a little more about your experience since mifepristone became available in Canada.

#	Question	Probe	Construct
1.	To begin, please tell me about your practice setting and your role in it.	Please tell me about your experience with mifepristone so far.	
		Have you begun offering mifepristone abortions? Why / Why not?	
		What is your past experience with abortion care? *(Probe for type of abortion services available.)*	Pre-existing knowledge skills, base (SA-AC)
		Can you describe your experience of getting information about how to implement mifepristone?	Knowledge seeking (AC)<br>Interpret / integrate knowledge (SA-AC)
		How was getting that information similar or different from how you learn about implementing other new drugs?	Learning style (AC)<br>Homophily (CI)
2.	**What was your experience like bringing mifepristone abortion into your facility?**<br>[For settings where it is not yet implemented, as what the participant thinks it WOULD be like for questions in section 2]	How did you / your setting make the decision to implement mifepristone? Who made the decision? What persuaded you?<br>*(How did you / your setting make the decision NOT to implement mifepristone?)*	Decentralization (SA-S)<br>Decision-Making (IP)<br>Marketing (CI)
		Have you observed or experience a gap in that mifepristone would fill?	Needs (AC)<br>Tension change (SR)
		How does (would) mifepristone abortion fit with your previous beliefs and practices?	Motivation (AC)<br>Values and goals (AC)

		How does (would) mifepristone abortion fit with your organization's previous beliefs and practices?	Homophily (CI)
		What are the expectations of your organization or professional college regarding implementation of mifepristone?	Inter-organizational norm-setting and networks (OC)
		Have there been (would there be) any unintended benefits to implementing mifepristone? Any unintended consequences? Please describe them.	Assessment of implications (SR)<br><br>Risk (I)
		Can you tell me about any political factors that have affected the implementation of mifepristone?<br><br>Have you experienced pressure to implement mifepristone? (If YES: Can you describe what happened?)	Socio-political climate (OC)
		How do (would) you use mifepristone? Have you modified (would you modify) the protocol in any way to fit your context?<br><br>What changes have you made (would you make) within your setting to implement mifepristone?	Innovation-system fit (SR)<br><br>Potential for reinvention (I)<br><br>Reinvention (IP)
		Can you describe the patient care pathway for mifepristone medical abortion in your setting (or what it would look like)? (*Probe for appointments, counseling rooms, prescriber relationships, dispensing, where and how each step takes place)*	Complex, nonlinear process (A)
		How does (would) mifepristone medical abortion impact your work? *(Probe for: Time, Resources, Professional relationships, Patients' ability to access abortion)*	Dedicated time and resources (SR)<br><br>Slack resources (SA-S)
3.	**What things make it challenging to provide mifepristone medical abortion?**	*Probe for the impact of the following factors*:<br>• Cost (such as provincial coverage, financial disincentives, uncertainty about coverage)<br>• Clinical workflow (such as counselling, following up; changing from a surgical to a medical abortion clinic)<br>• Documentation (such as Health Canada forms, consent forms)	Resource system (RS)<br><br>Environmental stability (OC)<br><br>Task issues (I)

		• Pharmacy stock (such as ordering it)<br>• Government support (such as political factors)<br>• Restrictions (such as physician dispensing)<br>• Anti-choice attitudes (such as among protestors or colleagues)<br>• Having community access to surgery, ultrasound, or labs<br>• Human resources (such as counsellors; staff burn out)	
		Have you learned about any changes to mifepristone regulations or coverage? What have you learned? Where did you get the information?	Reinvention (IP)<br>Complex, nonlinear process (A)<br>Fuzzy boundaries (I)
		What needs to change to make it easier to implement mifepristone?	Reinvention (IP)
		What are some of the <u>supports or structures</u> that would be helpful for implementing mifepristone?	Technical support (I)
		What kind of <u>human resources</u> do you need to support mifepristone medical abortion practice? (Do you have any gaps in human resources?)	Human resources issues (IP)
		What kind of <u>encouragement or incentives</u> have you encountered to implement mifepristone?	Incentives (OC)
		How comfortable are you with the uncertainty surrounding mifepristone? *(Probe for federal restrictions, costs, and coverage)*	Tolerance of ambiguity (AC)
4.	**In your practice setting, who is involved in implementing mifepristone? What are they doing to support it?**	How many others are involved in prescribing for abortions? What kind of care do they provide?<br>Do they recognize the need to adopt mifepristone practice?<br>Does/Did your organization have a formal plan for implementing it in your context? What does it look like?	Leadership and vision (SA-RC)
		Is there any individual or organization you would describe as <u>unsupportive</u>? What did they do?	Power balances (SR)
		How easy or hard is it to communicate with your pharmacy,	Differentiation (SA-S)

		hospital, or other clinicians about mifepristone?	
		Have you given feedback or spoken out about the implementation process?<br>Can you describe that feedback? [i.e. spoken to the media, contacted college registrar]	Monitoring feedback (SR)
		Have you exchanged information with anyone about mifepristone, either inside or outside of your organization?<br>What did that look like?	Knowledge sharing (SA-AC)<br>Internal/external communication (IP)<br>Social networks (CI)
		Have you consulted with anyone inside or outside your setting or an official expert regarding mifepristone or its implementation? What did you discuss?	Peer / expert opinion leader (CI)
		Were there key individuals that rallied to support and implement mifepristone? Can you describe what they did?	Champions / change agents (CI)<br>Hands-on approach by leaders (IP)
		Were there key individuals that connected you with other stakeholders? Can you describe what they did?	Boundary spanner (CI)
5.	**Tell me about the values and vision of your organization and how they relate to implementing mifepristone.**	Does everyone share the same values?	Clear goals and priorities (SA-RC)
		What are your relationships like between managers and staff in your organizations when it comes to implementing mifepristone?	Managerial relations (SA-RC)
		In your organization, how easy or hard is it to implement new treatments like mifepristone?	Risk-taking climate (SA-RC)
		Is there a formal process in your organization for making decisions and implementing new practices, like mifepristone?	Formalization (SA-S)
6.	**What are the advantages and**	How effective is it compared to other abortion methods? Do you	Relative advantage (I)

	**disadvantages of mifepristone as a new treatment?**	see there being any downsides to having mifepristone available in Canada?	
		How does dispensing mifepristone medical abortion fit in you're your current practice? (How does it work with the way care is currently delivered?)	Compatibility (I)
		How difficult is it to understand the steps involved in dispensing mifepristone medical abortion?	Complexity (I)
		How easy would it be to experiment with having mifepristone in your setting on a trial basis to see how it works?	Trialability (I)
		Have you observed any impact or improvement in your patient population since implementing mifepristone? Can you please describe it?	Observability (I)
		How will you know that you are achieving good outcomes with implementing mifepristone in your context? What kind of outcomes are you tracking?	High quality data capture (SA-RC)
**We are curious to know more about your experience with the SOGC mifepristone training program, too.**			
7.	**What was your experience of the training process?**	What made you decide to take the mifepristone training program?	Motivation (AC)<br>Values and goals (AC)
		How was the mifepristone training similar or different from how you learn about other new practices?	Homophily (CI)<br>Cosmopolitanism (AC)
		What is your preferred way of learning a new treatment?	Learning style (AC)
		How much of the training did you do?	Skills (AC)
		Were there elements that were more or less difficult to learn?	Knowledge seeking (AC)<br>Nature of knowledge (I)
		How easy or hard was it to put your training into practice?	Ability to find, interpret, re-codify, and integrate new knowledge (SA-AC)

		How could the training be improved?	
**We are also interested to learn about where you go for support with your medical abortion practice.**			
8.	**Are you a member of the Canadian Abortion Providers Support platform?**<br>**(If YES) Tell me your thoughts about it.**	(CAPS members) What do you like or dislike about the website? How can it be improved?<br>(Non-members) Have you heard about the website?<br>If NO, explain what the website is before following up: Would joining this website be useful for you? Why / why not?<br>If YES: To help us make it useful for you, what are some reasons why you haven't joined the website?	Social networks (CI)
		When you need information or advice about medical abortion, where else do you usually turn?	Social networks (CI)<br>Cosmopolitanism (AC)
**We are almost at the end of our questions. Through this study, we also want to understand why physicians and pharmacists might choose NOT to provide mifepristone.**			
10.	Is there anyone you recommend I talk to who has chosen not to take up medical abortion with mifepristone?	For instance, are there any physicians or pharmacists in your community who currently provide women's health care, but not abortion?	
11.	Is there anything else you think I should know?		
12.	Do you have any questions for me?		

**Additional items for consideration during analysis**	
Adopter's belief that events are under one's personal control (internal) or that events are largely a matter of chance or due to external events (external).<br>*We include a locus of control measure in the survey component of this mixed methods study*	Locus of control (AC)
Number and experience of providers; Date of program inception.	Size/maturity (SA-S)

In the survey component of this mixed methods study	Tenure (AC)
Same as “Monitoring and Feedback (SR)”	Feedback (IP)

Glossary

(I)	Innovation
(AC)	Adopter characteristics
(A)	Assimilation
(IP)	Implementation process
(CI)	Communication and influence
(SA-S)	System antecedents – Structure
(SA-AC)	System Antecedents – Absorptive Capacity for New Knowledge
(SA-RC)	System Antecedents – Receptive Context for Change
(SR)	System Readiness
(OC)	Outer Context
(RS)	Resource System
